# A Wide-Range TCSC Based ADN in Mountainous Areas Considering Hydropower-Photovoltaic-ESS Complementarity

**DOI:** 10.3390/s24186028

**Published:** 2024-09-18

**Authors:** Yao Guo, Shaorong Wang, Dezhi Chen

**Affiliations:** School of Electrical and Electronic Engineering, Huazhong University of Science and Technology, Wuhan 430074, China; d202080604@hust.edu.cn (Y.G.); dzhchen@hust.edu.cn (D.C.)

**Keywords:** TCSC, active distribution network, deep reinforcement learning, optimization operation

## Abstract

Due to the radial network structures, small cross-sectional lines, and light loads characteristic of existing AC distribution networks in mountainous areas, the development of active distribution networks (ADNs) in these regions has revealed significant issues with integrating distributed generation (DGs) and consuming renewable energy. Focusing on this issue, this paper proposes a wide-range thyristor-controlled series compensation (TCSC)-based ADN and presents a deep reinforcement learning (DRL)-based optimal operation strategy. This strategy takes into account the complementarity of hydropower, photovoltaic (PV) systems, and energy storage systems (ESSs) to enhance the capacity for consuming renewable energy. In the proposed ADN, a wide-range TCSC connects the sub-networks where PV and hydropower systems are located, with ESSs configured for each renewable energy generation. The designed wide-range TCSC allows for power reversal and improves power delivery efficiency, providing conditions for the optimization operation. The optimal operation issue is formulated as a Markov decision process (MDP) with continuous action space and solved using the twin delayed deep deterministic policy gradient (TD3) algorithm. The optimal objective is to maximize the consumption of renewable energy sources (RESs) and minimize line losses by coordinating the charging/discharging of ESSs with the operation mode of the TCSC. The simulation results demonstrate the effectiveness of the proposed method.

## 1. Introduction

Up to now, around the world, it has become a development trend to integrate renewable energy sources (RESs) in the form of distributed generations (DGs) into a traditional AC distribution network to form an active distribution network (ADN) [1,2]. This condition has led to dramatic alterations of distribution networks from their structures to operation control modes [3,4]. In the mountainous areas of southern China, RESs are quite affluent, such as hydro, solar, and wind energies, which have been or will be developed as run-of-river small hydropower (RoR-SH) stations, rooftop photovoltaic (PV) generation stations, and distributed wind farms. However, the existing AC distribution networks in these mountainous areas are almost radial and generally have the characteristics of small-section lines and light loads. With the increase in accessed DGs in these networks, the voltage violation issue is frequently emerging, the implementation of optimization operations is difficult, and partial transformers will operate under overload conditions while surplus DG power needs to feed the grid. Therefore, it is valuable and of practical significance to develop new link circuits with low-cost and optimization operation methods for integrating local RES generation systems into the AC distribution networks in the mountainous areas of southern China.

In the past decade or more, there has been significant attention on ADNs, with explosive growth in publications researching optimization operation strategies [5,6,7,8,9], energy storage system (ESS) configuration methods [10,11,12,13], new converter controllers [14,15,16], oscillation analysis, Volt-Var control [17,18,19,20], and other related areas. However, there has been very little focus on developing suitable and cost-effective structures specifically designed to enhance the capacity of ADNs in mountainous regions for integrating DGs, including both RoR-SH and PV.

Under the condition of considering the access of DGs, literature [21] proposed a method for planning the feeder automation configurations in the 10 kV distribution network of mountainous areas. Using the proposed method, the optimal recommendation table for determining the types and quantities of automatic switches for each kind of wiring can be obtained, and then the designed distribution network in mountainous areas is of higher reliability. However, the problems caused by accessing DGs remain unsolved. In [22], focusing on maximizing network utilization and mitigating power variations, an ADN connection equipment—a multi-terminal loop power flow controller combined by voltage source converters connected via a common DC link—is presented. The scheme can balance the loads among feeders, but it should be so expensive that it is not suitable to be used in ADNs of mountainous areas. In [23], a diamond-shaped distribution network structure is designed to improve power supply reliability and line capacity utilization in the current distribution network. The design is based on the typical wiring patterns in developed cities, e.g., Tokyo and Paris, and is also inapplicable to the wiring patterns of ADN in mountainous areas. So, until now, an ADN with excellent capacity for DG integration specifically designed for mountainous areas is wanted.

Motivated by the aforementioned facts, in this paper, we innovatively propose a wide-range thyristor-controlled series compensation (TCSC)-based ADN in mountainous areas, considering hydropower-photovoltaic-ESS complementarity. The proposed ADN incorporates which includes RoR-SH stations, rooftop PV generation stations, and ESSs. Also, a corresponding deep reinforcement learning (DRL)-based optimal operation strategy for the proposed ADN is designed in detail. This paper mainly achieves the following contributions:

(1) At the equipment level, a wide-range TCSC is designed to connect sub-networks in ADNs in mountainous areas, where RoR-SH stations and rooftop PV generation stations are located. Unlike traditional TCSCs, which are primarily set to capacitive mode for transient stability control of power systems, the proposed wide-range TCSC can be continuously adjusted for a larger range of impedance values. This design avoids the issues of resonance that can occur when the TCSC is regulated near its boundary values. Moreover, the designed wide-range TCSC is intended to enhance power transfer efficiency and facilitate power inversion during steady-state operation, providing essential support for the proposed optimal operation strategy for the ADN in mountainous areas.

(2) At the system level, with the goal of achieving the maximum consumption of renewable energy and minimum line losses, further reducing curtailment of solar and hydropower as well as the issue of voltage violation in the ADNs of mountainous areas, a DRL-based optimal operation strategy is proposed considering hydropower-photovoltaic-ESS complementarity.

A Markov decision process (MDP) formulation Is built for the optimal operation problem considering the coordinated control of ESSs and the wide-range TCSC to achieve RES efficient consumption. Unlike the existing math methods, the learned operation strategy based on MDP can deal with the variables of continuous space and achieve superior precision control performance.

(3) By coordinating the application of the designed wide-range TCSC with the proposed optimal operation strategy that considers the complementarity of solar, hydropower, and ESS, the integration capacity of renewable energy generation in mountainous distribution networks can be significantly improved. This approach effectively addresses current issues such as poor transmission efficiency and voltage violations in mountainous distribution networks, thereby demonstrating practical value.

The remainder of the paper is structured as follows: Section 2 describes the principle of the wide-range TCSC and introduces the topology of the proposed wide-range TCSC-based ADN. Section 3 illustrates the DRL-based hydropower-photovoltaic-ESS complementarity optimal operation strategy in detail. In Section 4, simulations are provided to show the effectiveness of the strategy. Section 5 draws the conclusion.

## 2. Principle of Wide-Range TCSC

TCSC is an important piece of equipment in the flexible AC transmission system due to its multi-functions such as power flow control, damping low-frequency power oscillation, improving transient stability, and suppressing sub-synchronous resonance. However, in this paper, a wide-range TCSC is designed to expand the function of TCSC from power flow control to the range of power flow reversal.

In this section, the principle of the designed wide-range TCSC used in the proposed ADN is introduced, and the topology of the proposed wide-range TCSC-based is introduced.

### 2.1. Wide-Range TCSC Operation Principle

**Figure 1:**
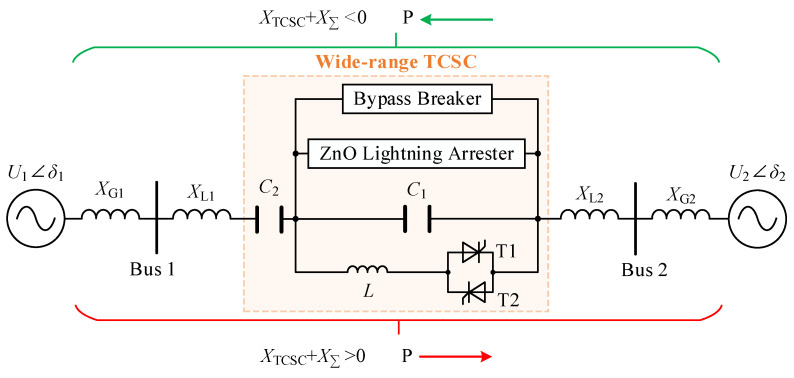
The diagram of the wide-range TCSC.

The wide-range TCSC consists of a traditional TCSC in series with a group of capacitors and includes six main parts: two groups of capacitors, one reactor, one group of bi-directional thyristors, one ZnO lighting arrester, and one bypass breaker, as shown in Figure 1. In this case, the positive or negative of the sum of the reactances $X_{\mathrm{TCSC}}+X_{\Sigma }$ between the two sources determines the flow of power.

**Figure 2:**
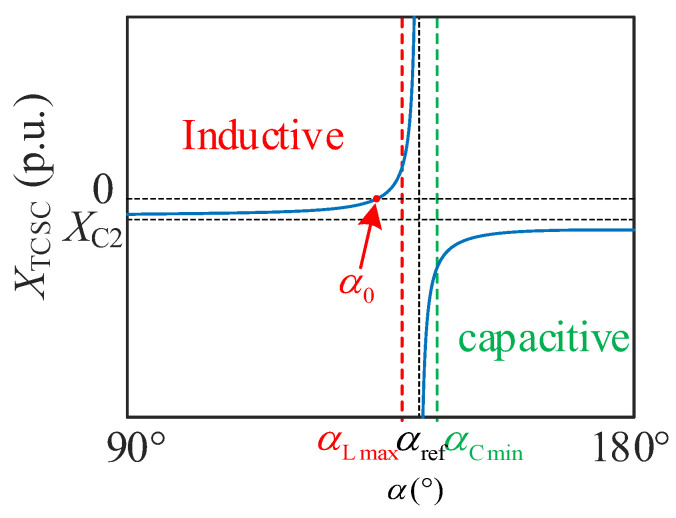
The change curve of $X_{\mathrm{TCSC}}$ with α under the industrial frequency.

Obviously, by controlling the trigger angle of the thyristors, the equivalent reactance $X_{\mathrm{TCSC}}$ of the wide-range TCSC can be changed within a certain inductive and capacitive range. $X_{\mathrm{TCSC}}$ can be expressed as follows:

(1) $$\left\{\begin{array}{l} X_{\mathrm{TCSC}}=-\frac{1}{\omega C_{1}}-\frac{1}{\omega C_{2}}+\frac{A}{\pi \omega C_{1}}\left[2\left(\pi -\alpha \right)+\sin\left(2\left(\pi -\alpha \right)\right)\right]-\frac{4A}{\pi \omega C_{1}\left(k^{2}-1\right)}\cos^{2}\left(\pi -\alpha \right)*\eta \\ \omega_{0}=\sqrt{\frac{1}{LC_{1}}}\\ A=\frac{\omega_{0}^{2}}{\omega_{0}^{2}-\omega^{2}}\\ k=\frac{\omega_{0}}{\omega }\\ \eta =\left[k\tan\left(k\left(\pi -\alpha \right)\right)-\tan\left(\pi -\alpha \right)\right] \end{array}\right.$$

where ω is the angular frequency under the practical operation condition, $\alpha \in \left[\pi /2,\pi \right]$ is the trigger angle of thyristors. The change curve of $X_{\mathrm{TCSC}}$ with α under the industrial frequency is shown in Figure 2. Wherein, $\alpha_{0}$ and $\alpha_{\mathrm{ref}}$ are the trigger angles corresponding to when $X_{\mathrm{TCSC}}=0$ and when the wide-range TCSC generates resonance, respectively.

When $\alpha \in \left[\alpha_{0},\alpha_{\mathrm{ref}}\right)$, the wide-range TCSC operates in adjustable inductive mode. And it presents a capacitive equivalent reactance when $\alpha \in \left[90{}^{\circ},\alpha_{0}\right]\cup \left(\alpha_{\mathrm{ref}},180{}^{\circ}\right]$ (named adjustable capacitive mode). Under the block mode of α=180°, $X_{\mathrm{TCSC}}$ is equal to value $X_{C1}+X_{C2}$. In the bypass mode of α=90°, $X_{\mathrm{TCSC}}$ is equal to $X_{C2}$ plus the reactance value of paralleling the capacitor and reactor.

Because the excessively high voltage between the two ports of the traditional TCSC will occur when α is close to $\alpha_{\mathrm{ref}}$, α must be limited within the range $\left[90{}^{\circ},\alpha_{\mathrm{Lmax}}\right)\cup \left(\alpha_{C\min},180{}^{\circ}\right]$.

In this paper, the wide-range TCSC plays the role of adjusting and even reversing power flow. As shown in Figure 2, the active power *P* flowing from Bus 1 to Bus 2 can be expressed as follows:

(2) $$\left\{\begin{array}{l} P=\frac{U_{1}U_{2}}{X_{\Sigma }+X_{\mathrm{TCSC}}}\sin\left(\delta_{1}-\delta_{2}\right)\\ X_{\Sigma }=X_{G1}+X_{G2}+X_{L1}+X_{L1} \end{array}\right..$$

**Figure 3:**
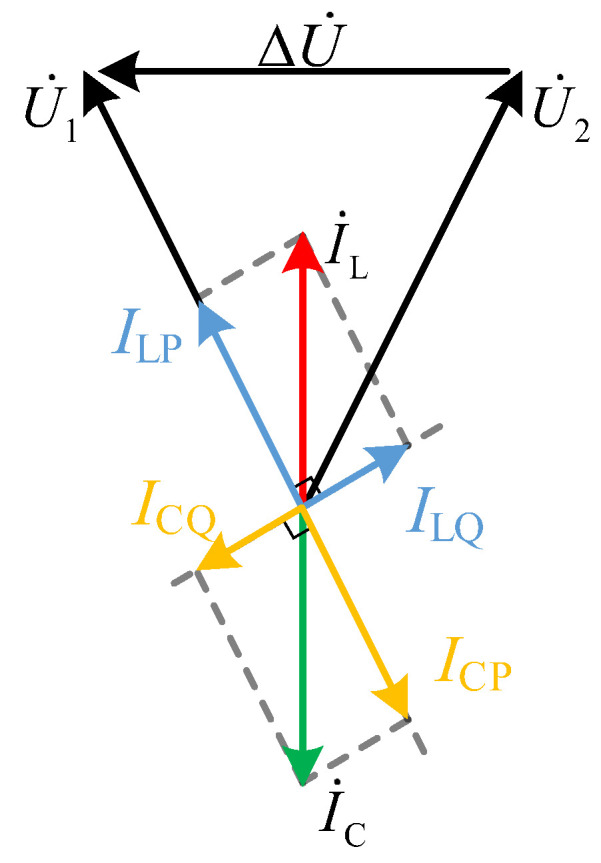
The power-voltage phase relationship diagram.

And, the power-voltage phase relationship in Figure 1 is shown in Figure 3.

It can be seen that the magnitude and direction of *P* can be adjusted by changing the reactance value $X_{\mathrm{TCSC}}$ of the wide-range TCSC, which is determined by α.

### 2.2. Topology of Wide-Range TCSC-Based ADN

**Figure 4:**
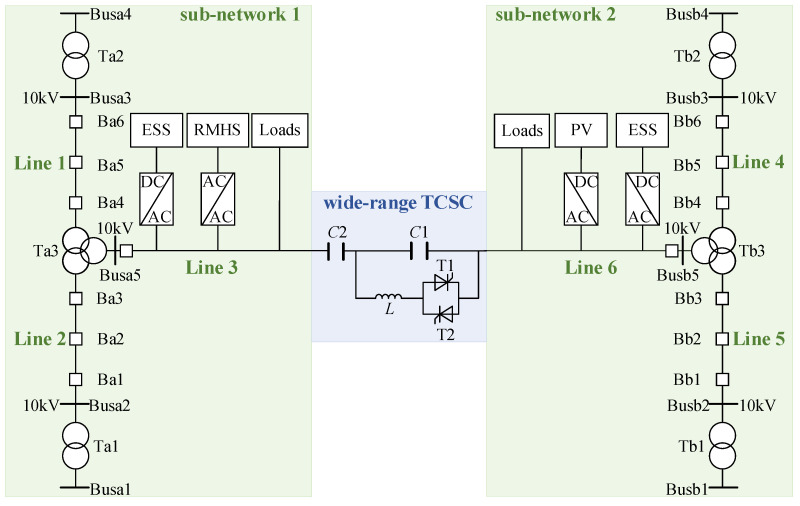
The topology of the proposed wide-range TCSC-based ADN.

Figure 4 demonstrates the proposed wide-range TCSC-based and in the mountainous area, which includes two T-shaped sub-networks and a wide-range TCSC.

In each T-shaped sub-network, three 10 kV AC lines are linked together through a three-winding transformer. Wherein the ends of two AC lines are respectively connected to two 10 kV buses that belong to the substations of the 110 kV/220 kV main grid. And branches of the above two AC lines can be used for serving power users. The remaining AC line is used to connect the wide-range TCSC for the connection between two sub-networks. Meanwhile, it also takes charge of integrating DGs, ESSs, and loads. As shown in Figure 4, this paper studies the case that RoR-SHs and PVs are severally integrated in sub-network 1 and in sub-network 2. Also, both Line 3 and Line 6 are equipped with respective ESS.

Traditional TCSC always operates in the capacitive mode to improve power system stability. But it is also theoretically possible to achieve the power reversal on the line with the traditional one. However, due to the limitation of the boundary value $\alpha_{C\min}$ of the trigger angle, the maximum absolute value of the capacitive $X_{\mathrm{TCSC}}$ may be smaller than the inductive equivalent impedance value $X_{\Sigma }$, resulting in the impossibility of power reversal. Thus, the proposed wider range of TCSC reactance is needed.

**Figure 5:**
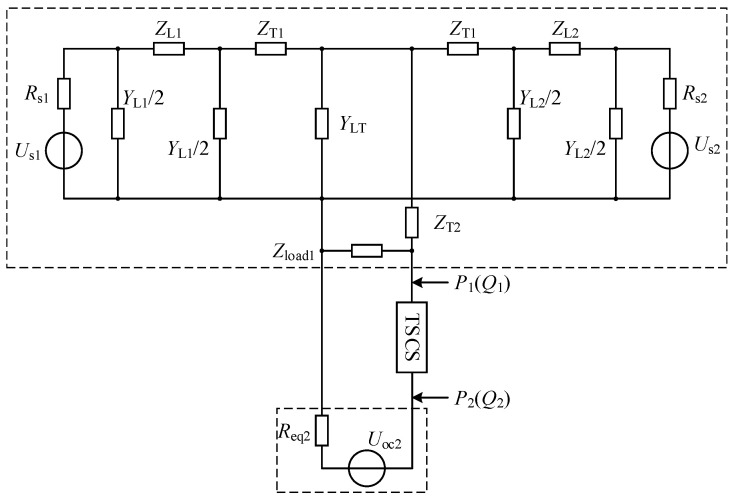
Thevenin equivalent circuit of the proposed ADN.

According to Thevenin’s theorem, the proposed ADN of Figure 4 can be equated to a double-generator power system as shown in Figure 5. Therefore, the proposed wide-range TCSC analysis can be applied to the proposed ADN.

The open circuit voltage $U_{\mathrm{oc}1}$, $U_{\mathrm{oc}2}$ and equivalent resistance $R_{\mathrm{eq}1}$, $R_{\mathrm{eq}2}$ can be calculated as follows:

(3) $$\left\{\begin{array}{cc} \; & f\left(x,y\right)=\frac{xy}{x+y}\\ \; & X_{n}=\frac{\left(f\left(R_{Sn},Y_{Ln}/2\;\right)+Z_{Ln}\right)\times Y_{Ln}/2\;}{f\left(R_{Sn},Y_{Ln}/2\;\right)+Z_{Ln}+Y_{Ln}/2\;}+Z_{T1},\begin{array}{cc} & n\in \left\{1,2,3,4\right\} \end{array}\\ \; & Z_{m}=\frac{X_{2m-1}X_{2m}Y_{\mathrm{LT}}}{X_{2m-1}Y_{\mathrm{LT}}+X_{2m}Y_{\mathrm{LT}}+X_{2m-1}X_{2m}}+Z_{T2},\begin{array}{cc} & m\in \left\{1,2\right\} \end{array}\\ \; & R_{om}=\frac{Z_{m}Z_{\mathrm{load}m}}{Z_{m}+Z_{\mathrm{load}m}}\\ \; & X\left(a,c\right)=\frac{f\left(f\left(X_{a},Y_{\mathrm{LT}}\right),Z_{T2}+Z_{\mathrm{load}c}\right)}{Z_{T2}+Z_{\mathrm{load}c}},\begin{array}{cc} & a\in \left\{1,2\right\} \end{array}\begin{array}{cc} & c\in \left\{1,2\right\} \end{array}\\ \; & X\left(a,b,c\right)=f\left(X\left(a,c\right)\left(Z_{T2}+Z_{\mathrm{load}c}\right)+Z_{T1},Y_{Lb}/2\;\right)+Z_{Lb},\begin{array}{cc} & b\in \left\{1,2\right\} \end{array}\\ \; & R_{\mathrm{eq}c}=X\left(a,b,c\right)\\ \; & U_{\mathrm{oc}}\left(a,b,c\right)=\frac{Z_{\mathrm{load}c}U_{sb}f\left(X\left(a,b,c\right),Y_{Lb}/2\;\right)}{X\left(a,b,c\right)\left(R_{s2}+f\left(X\left(a,b,c\right),Y_{Lb}/2\;\right)\right)}\times \frac{\left(X\left(a,b,c\right)-Z_{Lb}\right)}{\left(Z_{T2}+Z_{\mathrm{load}c}\right)}\\ \; & U_{\mathrm{oc}1}=U_{\mathrm{oc}}\left(1,2,1\right)+U_{\mathrm{oc}}\left(2,1,1\right)\\ \; & U_{\mathrm{oc}2}=U_{\mathrm{oc}}\left(3,4,2\right)+U_{\mathrm{oc}}\left(4,3,2\right) \end{array}\right..$$

### 2.3. The Feasibility Verification of Wide-Range TCSC

The feasibility of the wide-range TCSC will be verified in two cases. One is from the adjustable inductive mode regulating to the adjustable capacitive mode; the other is the opposite. Wherein, in the former case, it is required that the wide-range TCSC is firstly controlled to switch to the bypass mode and then to the block mode for the alteration. For the second transition case, the switch procedure is the reverse of the above.

However, a fast and direct TCSC control method, especially one that results in a conversion of the power flow direction in an AC line, will definitely cause violent oscillation or even the destabilization of the power system. Therefore, a smooth TCSC control strategy for the proposed is essential. In this simulation, an equal interval transformation method is adopted to change the impedance of TCSC each time. When it is necessary to adjust tande impedance value of the TCSC from $X_{\mathrm{TCSC}1}$ to $X_{\mathrm{TCSC}2}$, set an interval of $\left(X_{\mathrm{TCSC}1}-X_{\mathrm{TCSC}2}\right)/n$ to be adjusted every 0.02 s, where *n* = 10 in the simulation cases of this section.

In these cases, the parameters of the propoand ADN are listed in Table 1. The network parameters are selected with reference to the actual distribution network. The trigger angles $\alpha_{L}$ and $\alpha_{C}$ corresponding to the two modes are randomly selected.

In case 1, with the initial power flow from sub-network 1 to sub-network 2, the initial trigger angle α of the wide-range TCSC is 132°, at which time $X_{\mathrm{TCSC}}=1.7708\Omega$. Since 0.3 s, by adjusting α, the impedance value of the TCSC is controlled to reduce by roughly 3 Ω every 0.02 s. The adjustment of α stops at 0.48 s, when $X_{\mathrm{TCSC}}=-27.3106\Omega$, corresponding to α of 145.5°.

**Figure 6:**
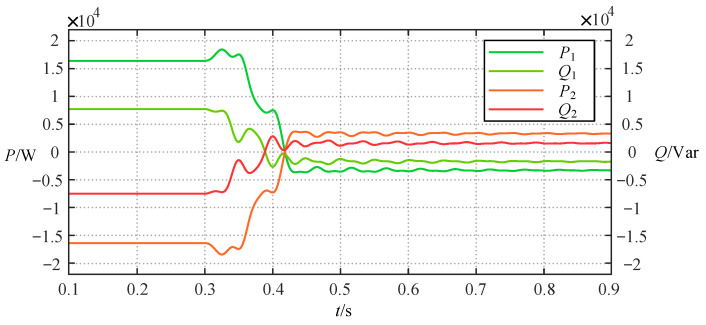
The change curve of the active and reactive power on both sides of the TCSC in case 1.

**Figure 7:**
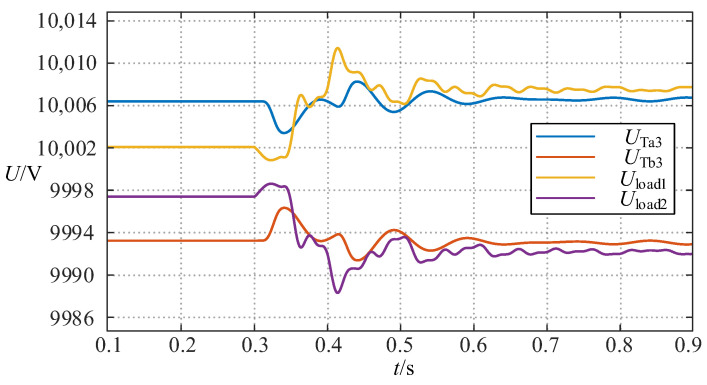
The change curve of several voltages in case 1.

The simulation result of the above process is shown in Figure 6, in which *P*_1_ *Q*_1_ and *P*_2_ *Q*_2_ are the active and reactive power on the left and right sides of the TCSC, respectively. It can be seen that the wide-range TCSC functions of power inversion can be well implemented. Figure 7 proves that the variations of the load voltage *U*_load1_ and *U*_load2_ on the line where the TCSC is located and the voltage *U*_Ta3_ and *U*_Tb3_ on the third winding sides of the three-winding transformers are always within the permissible voltage fluctuation range during the above process.

**Figure 8:**
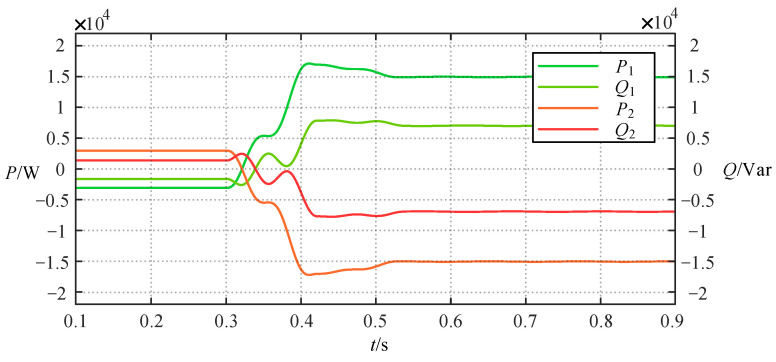
The change curve of the active and reactive power on both sides of the TCSC in case 2.

**Figure 9:**
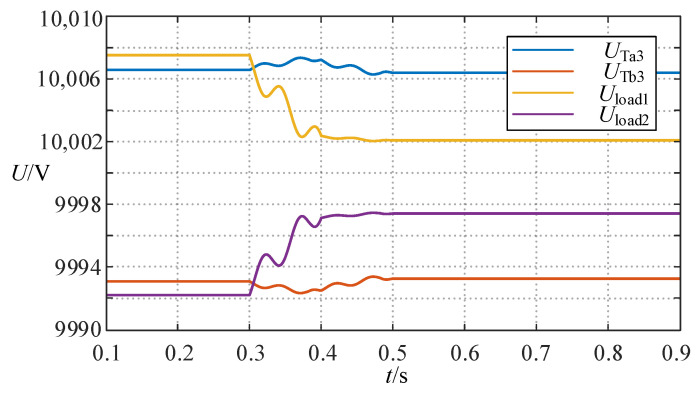
The change curve of several voltages in case 2.

For case 2, the initial power flow from sub-network 2 to sub-network 1, α is adjusted from 145.5° to 132° using the same method described above, resulting in the equivalent reactance value of the TCSC being adjusted from −27.3106 Ω to 1.7708 Ω. Figure 8 verifies the success of the power reversal, and Figure 9 demonstrates that this process does not suffer from the voltage violation.

## 3. DRL-Based Optimization Operation Strategy

This section proposes a DRL-based optimization operation strategy considering hydropower-photovoltaic-ESS complementarity for the proposed ADN. Firstly, the optimization problem is described in general. Then, we formulate the optimization control problem of ADN as a MDP. TD3 algorithm is introduced to solve the problem.

### 3.1. Problem Description

It is well known that in the distribution network of mountainous areas, RoR-SH provides stable power but cannot regulate its output and relies entirely on natural runoff. On the other hand, PV generation is intermittent and unstable, as its output is affected by sunlight intensity, weather conditions, and seasonal changes. In a considering hydropower-photovoltaic-ESS complementary system, hydropower provides stable power, PV generation offers additional peak power, and the energy storage system balances the fluctuations between the two, ensuring a stable power supply while reducing dependence on traditional fossil fuels.

In order to reduce light and water waste and promote the consumption of renewable energy, a DRL-based optimization operation strategy with hydropower-photovoltaic-ESS complementarity for the proposed ADN is proposed in this paper. The strategy aims to make the two types of DGs (i.e., RoR-SH and PV) with intermittent characteristic compensate each other in terms of power generation by controlling the wide-range TCSC and ESSs in multiple scenes, thus maximizing the local consumption of DG’s power and minimizing line losses. Wherein the former can be viewed as minimizing the supply of energy from the main grids to the ADN. Then, the objective function can be expressed as follows:

(4) $$\max f=-\left(\sum_{n}^{N=4}P_{n}+{\sum P}_{i}^{\mathrm{loss}}\right)$$

where $\sum_{n}^{N=4}P_{n}$ are the active power supplied by the main grid *n* in the proposed ADN, in other words, they are the active power on Busa1, Busa4, Busb1, and Busb4 of the proposed ADN, respectively; ${\sum P}_{i}^{\mathrm{loss}}$ represents the active power loss in line *i*.

### 3.2. MDP Formulation of the Optimization Operation Strategy

The problem of the optimization operation strategy can be formulated as an MDP, which is defined as a tuple {***S***, ***A***, ***P***, ***R***, γ}, where ***S*** is a set of the environment states, ***A*** is the action set, ***P*** is the state transition probability, ***R*** is the immediate reward set, and γ is the discount factor of the reward. The definitions of State *s_t_*, Action *a_t_*, Reward *r_t_*, and probability *p* at *t* time-step are elaborated as follows:

1. **State**: In the environment formed by the proposed ADN, state space *s_t_* can be defined as follows:
  (5) $$s_{t}=\left(P_{t}^{\mathrm{PV}},P_{t}^{\mathrm{RMHS}},P_{t}^{\mathrm{load}},P_{1,t}^{\mathrm{ESS}},P_{2,t}^{\mathrm{ESS}},X_{1,t}^{\mathrm{SOC}},X_{2,t}^{\mathrm{SOC}},\alpha_{t}^{\mathrm{TCSC}},X_{t}^{\mathrm{TCSC}},P_{1,t}^{\mathrm{grid}},P_{2,t}^{grid},P_{3,t}^{\mathrm{grid}},P_{4,t}^{\mathrm{grid}}\right)$$
  where $P_{t}^{\mathrm{PV}}$, $P_{t}^{\mathrm{RMHS}}$, and $P_{t}^{\mathrm{load}}$ denote the active power generated by the PV and RoR-SH, and demanded by the users; $P_{1,t}^{\mathrm{ESS}}$ and $P_{2,t}^{\mathrm{ESS}}$, $X_{1,t}^{\mathrm{SOC}}$ and $X_{2,t}^{\mathrm{SOC}}$ are the active power outputs and the states of charge (SOCs) of the ESSs; $\alpha_{t}^{\mathrm{TCSC}}$ and $X_{t}^{\mathrm{TCSC}}$ present the trigger angle and the equivalent reactance value of the wide-range TCSC, respectively.
2. **Action**: The action $a_{t}$ represents the setting value of the trigger angle $\alpha_{t}^{\mathrm{TCSC}}$ of the wide-range TCSC and the active power $P_{1,t}^{\mathrm{ESS}}$ and $P_{2,t}^{\mathrm{ESS}}$ charged/discharged by the ESSs at timeslot *t* with the given state $s_{t}$. Action $a_{t}$ can be present as follows:
  (6) $$a_{t}=\left(\alpha_{t}^{\mathrm{TCSC}},P_{1,t}^{\mathrm{ESS}},P_{2,t}^{\mathrm{ESS}}\right).$$
  wherein, $P_{1,t}^{\mathrm{ESS}}$ and $P_{2,t}^{\mathrm{ESS}}$ are both limited by the maximum and minimum value of the charge/discharge of the ESSs. Due to the wide-range TCSC operation principle in Section 2, $\alpha_{t}^{\mathrm{TCSC}}$ is restricted as follows:
  (7) $$\alpha_{t}^{\mathrm{TCSC}}\in \left[90{}^{\circ},\alpha_{\mathrm{Lmax}}\right)\cup \left(\alpha_{C\min},180{}^{\circ}\right].$$
3. **Reward**: In accordance with the optimization operation objective of maximizing the local consumption of renewable energy and minimizing line losses analyzed in Section 3.1, the reward $r_{t}$ can be defined as follows:
  (8) $$r_{t}=-\left(\sum_{n=1}^{4}P_{n}+{\sum P}_{i}^{\mathrm{loss}}\right)-k$$
  where *k* is a penalty term that can be used to correct an improper action strategy. For the proposed ADN, the current action may cause the SOCs of the ESSs in the next state to exceed their permissible upper and lower limits which is not allowed. Meanwhile, voltage violation is not permitted. Thus, *k* is set to be the following:
  (9) $$\left\{\begin{array}{l} k_{1}=\left\{\begin{array}{l} X_{t}^{\mathrm{SOC}}-X_{t}^{\mathrm{SOCmax}}\begin{array}{cc} & X_{t}^{\mathrm{SOCmax}}<X_{t}^{\mathrm{SOC}} \end{array}\\ X_{t}^{\mathrm{SOCmin}}-X_{t}^{\mathrm{SOC}}\begin{array}{cc} & X_{t}^{\mathrm{SOC}}\leq X_{t}^{\mathrm{SOCmin}} \end{array} \end{array}\right.\\ k_{2}=\left\{\begin{array}{l} U_{i,t}-1.05U_{Ni}\begin{array}{cc} & 1.05U_{Ni}<U_{i,t} \end{array}\\ 0.95U_{Ni}-U_{i,t}\begin{array}{cc} & U_{i,t}\leq 0.95U_{Ni} \end{array} \end{array}\right.\\ k=p\left(k_{1}\right)+q\left(k_{2}\right) \end{array}\right.$$
  where *p* and *q* are the penalty factor; $X_{t}^{\mathrm{SOCmax}}$ and $X_{t}^{\mathrm{SOCmin}}$ are the maximum and minimum SOC values specified for the ESSs, respectively; $U_{i,t}$ is the voltage at node *i* in the ADN, whose permissible deviation is ±5% of the rated voltage $U_{Ni}$.
4. **State Transition**: In the current state $s_{t}$, the trigger angle α of the wide-range TCSC and the active power charged/discharged by the ESSs are controlled according to the action strategy $a_{t}$, which refreshes the power flow of the proposed ADN and makes the node voltages fluctuate randomly. Thus, the state of the next moment $s_{t+1}$ can be obtained. The probability *p* of the process of $s_{t}$ transited to $s_{t+1}$ is as follows:
  (10) $$p\left(s_{t},s_{t+1}\right)=\mathrm{Pr}\left(s_{t+1}\left|s_{t},a_{t}\right.\right).$$

### 3.3. Twin Delayed Deep Deterministic Policy Gradient

Based on the above MDP model, the above hydropower-photovoltaic-ESS complementarity optimization operation strategy model can be transformed into a DRL framework so that it can be solved using DRL algorithms. In this section, we introduce a TD3 algorithm, which is an advanced policy gradient algorithm for solving continuous action control issues, to solve the optimization operation problem of the proposed ADN.

**Figure 10:**
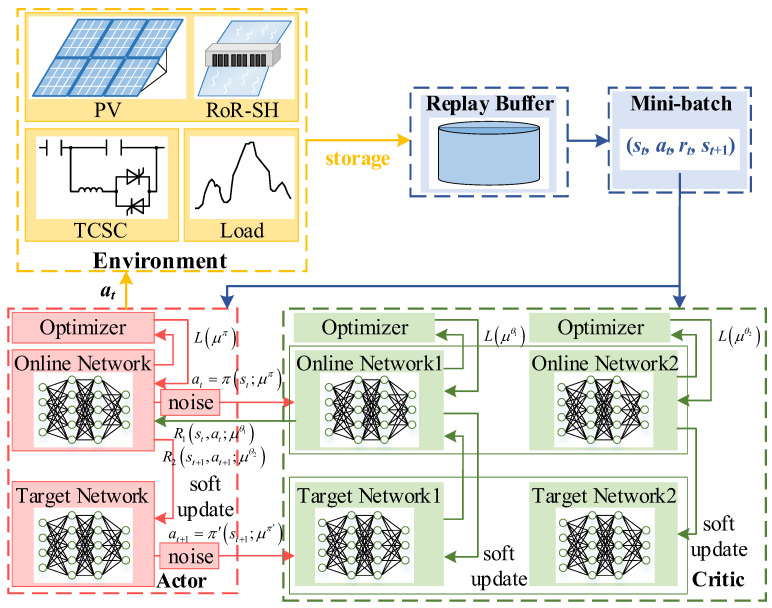
TD3 training architecture diagram.

The TD3 algorithm contains a total of six neural networks, which are divided into two main categories: one pair of Actor Online-Target networks π and two pairs of Critic Online-Target networks $\theta_{1}$ and $\theta_{2}$. Actor networks can directly select action value by the policy in continuous space, while Critic networks can judge the behavior of the Actor networks under the current state by fitting the action value function. Its training architecture diagram is shown in Figure 10.

During the learning process, the proposed ADN state $s_{t}$ is extracted and fed into the Actor online network, which will give the corresponding action $a_{t}$ and interact with the environment once. The action $a_{t}$ at time *t* can be expressed as $a_{t}=\pi \left(s_{t};\mu^{\pi }\right)$, where $\mu^{\pi }$ is the weight of the Actor network. Then, a new action instruction $a_{t+1}$ will be given according to the new state $s_{t+1}$. Noise can be added to the action during the training process. After an interaction is completed, the experience replay buffer stores the interaction information, and the network parameters will be updated by loss functions *L* when the interaction information stored in the experience replay buffer exceeds the number of network parameter update batches *B*. The Actor optimizer and the Critic optimizer update the Actor online network parameters and the Critic online network parameters based on the sampled data, respectively, and the Actor target network and the Critic target network are soft updated based on the current Actor online network as well as the Critic online network parameters. When the interaction information stored in the experience replay buffer exceeds the capacity, the initial interaction data are deleted.

DRL algorithms always can be divided into three categories: value-based algorithm, policy-based algorithm, and Actor-Critic algorithm, and it has been verified that the Actor-Critic algorithm always has better results. TD3 is a deterministic policy gradient algorithm with an Actor-Critic frame. It combines Deep Deterministic Policy Gradient (DDPG) and double networks, which can improve DDPG. On the basis of DDPG, TD3 adopts three pivotal technologies.

**Figure 11:**
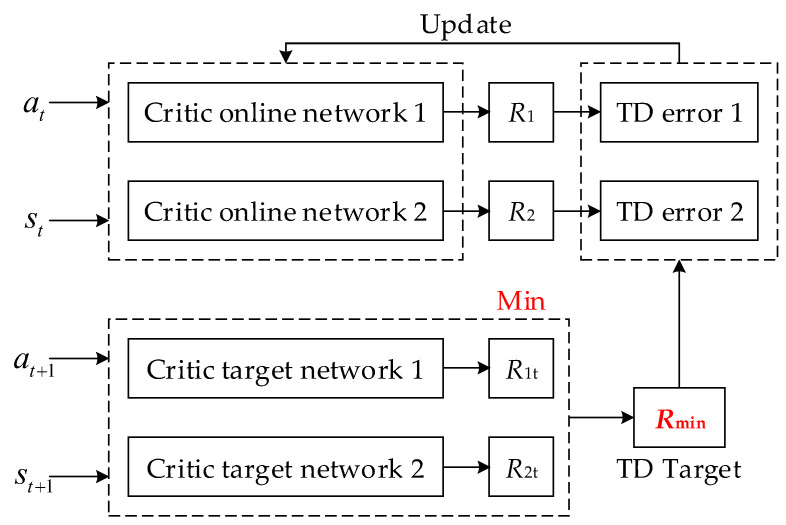
Double networks update.

1. **Double Networks**: Essentially, the TD3 algorithm is a method of Actor-Critic. What is distinct is that TD3 has two Critic online networks and two Critic target networks. The two pairs of Critic Online-Target networks calculate the *R*-values at the same time as the parameter update, but the smaller *R*-value (TD Target) is selected for updating the Crttic networks, as shown in Figure 11.

This method can effectively avoid producing the problem of overestimation caused by the calculation of maximization in DDPG.

2. **Target Policy Smoothing Regularization**: When the Critic network is updated in DDPG, the learning process with the deterministic policy easily occurs overfitting phenomenon, which results in a large variance of the target estimation and inaccurate estimation value. This problem can be reduced by regularization, so that target policy smoothing, a regularization method, is introduced. When the target value is calculated, noise obeying a normal distribution is added to the action value to improve the accuracy of the target value estimation and to ensure the stability of the network training process. The amended TD Target can be calculated by the following:
  (11) $$\begin{array}{cc} \; & y=r+\gamma \min_{i=1,2}R_{i}\left(s_{t+1},\pi_{t+1}\left(s_{t+1}\right)+\varepsilon \right)\\ \; & \varepsilon ∼\mathrm{clip}\left(N\left(0,\tilde{\sigma }\right),-c,c\right) \end{array}$$
  where γ is the discount factor; ε is the clipped noise, which is restricted in [-*c*, *c*] and follows a normal distribution.

3. **Delayed Update**: After the Critic network has been updated several times, the Actor network will be updated once, so as to ensure the training of the Actor network is more stable.

## 4. Simulations

In this section, we evaluate the performance of the TD3-based optimization operation strategy with hydropower-photovoltaic-ESS Complementarity for the proposed ADN.

### 4.1. Experimental Setup

The simulation is conducted based on the ADN shown in Figure 4. The generation information for PV and RoR-SH and load demand information are derived from real-time series data downloaded from a power system research institution in a province in southern China. The data spans from 1 January 2020 for a duration of 3 years, with recordings taken at hourly intervals. Data from the first 20 days of each month are used for training, while the remaining days serve as the test set. The data are scaled appropriately for the system under consideration. Additionally, network parameters and wide-range TCSC parameters follow the specifications in Table 1. The ESSs have the same capacity of 3 MVA and a regulation range of [−0.1, 0.1] MVA.

A training episode is configured to last 24 h, with each hour representing a step, resulting in a total of 24 steps per episode. After each action is executed, feedback in the form of a reward is received from the environment. The episode concludes normally after running successfully for 24 h. However, if SOCs of ESSs or node voltages exceed their limits during any step, a large negative reward is given, and the episode is terminated immediately, after which a new training episode begins.

To evaluate the advantages of the proposed strategy in terms of renewable energy consumption capacity, four cases are set up for comparative analysis:

1. **Case 1:** Select data from May 15, which represent a period of abundant water with lower PV generation;
2. **Case 2:** Select data from November 15, which correspond to the period of reduced water availability and higher PV generation;
3. **Case 3:** Select data from May 15 and employ the ADN without ESSs;
4. **Case 4:** Select data from May 15 and employ the ADN without wide-range TCSC;
5. **Case 5:** Select data from May 15 and employ the ADN without wide-range TCSC and ESSs.

**Figure 12:**
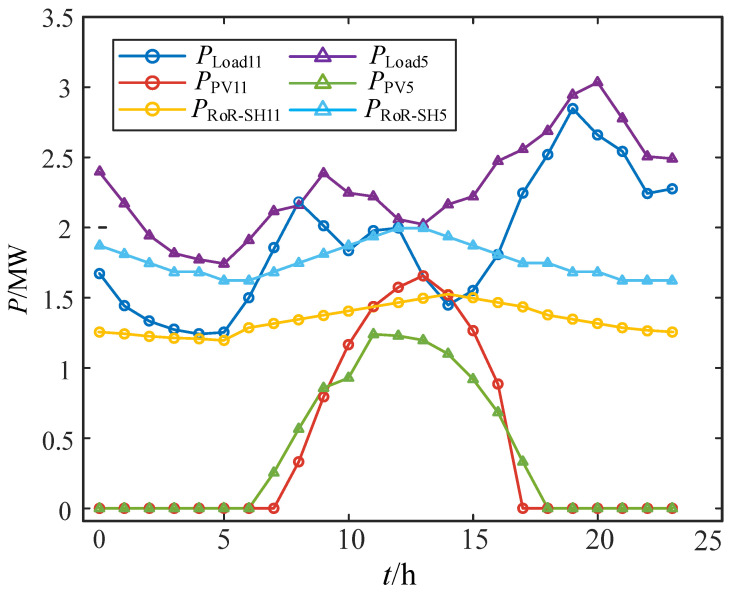
Information on renewable energy and load.

The information on renewable energy and load on the above two days is shown in Figure 12.

### 4.2. Simulation Result Analysis

#### 4.2.1. Algorithm Performance

**Figure 13:**
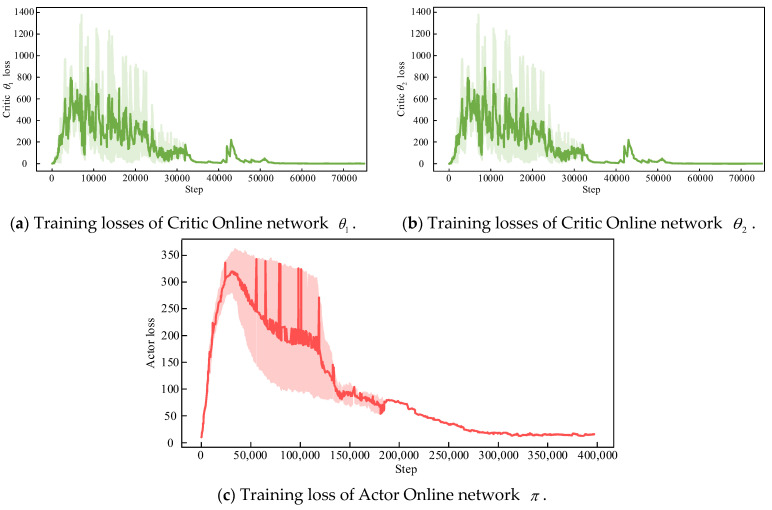
TD3 training losses.

The loss functions of the three neural networks, the Critic Online network $\theta_{1}$ and $\theta_{2}$, and the Actor Online network π that need to be trained in TD3, are shown in Figure 13. The dark curve is the result of smoothing the true light curve. It can be seen that these training loss values are towards convergence after several steps, indicating that good value judgments and action decisions have been learned to make.

**Figure 14:**
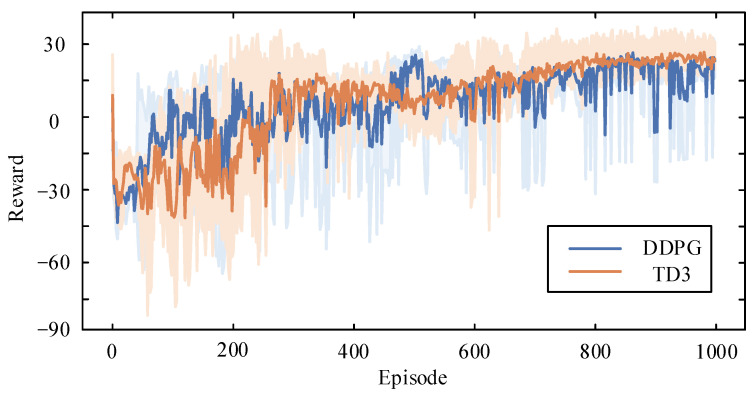
Rewards comparison between two DRL algorithms.

In Figure 14, the training rewards are depicted, with the light-colored curves representing the real reward values under random initial value settings of network parameters, while the darker curve shows the average reward per episode. Initially, the rewards per episode are low, indicating that the training is receiving negative rewards and episodes are cut short. This early phase reflects the DRL algorithms’ exploration of the environment and the lack of effective optimization strategies. As training progresses, the algorithm improves through interaction with the environment, leading to longer episode durations. After 600 episodes, the duration of each episode reaches 24 h with minimal fluctuations, demonstrating that effective strategies have been learned to maintain safe and stable operation without exceeding limits. It can also be seen from Figure 14 that the TD3 algorithm used in this paper achieves more convergent results and higher reward values compared to the DDPG algorithm.

#### 4.2.2. ESSs and Wide-Range TCSC Control Results

**Figure 15:**
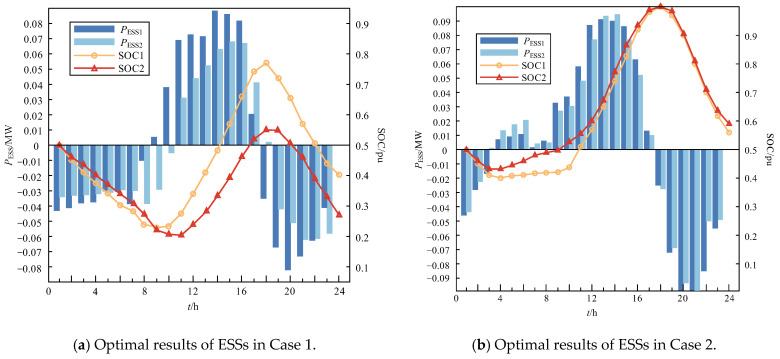
Active power outputs and SOCs of ESS curve.

**Figure 16:**
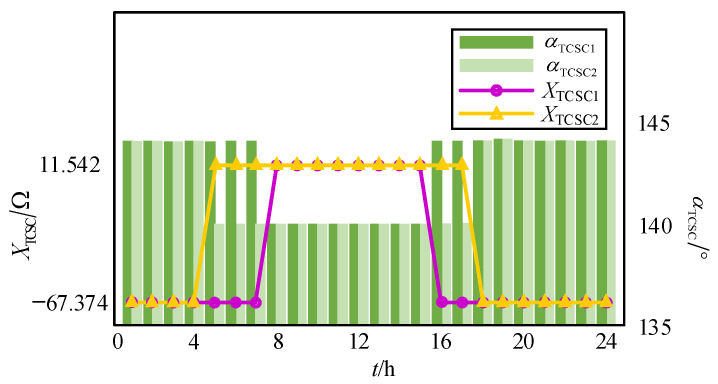
Optimal results of the wide-range TCSC in Case 1 and Case 2.

The optimization results over a 24 h period are shown in Figure 15 and Figure 16. Specifically, Figure 15a,b display the active power outputs and SOCs of the ESSs for Case 1 and Case 2, respectively, and Figure 16 illustrates the trigger angle and equivalent impedance variation of the wide-range TCSC for both cases.

From Figure 15, it can be seen that in the period of abundant water, during the period of 0:00–2:00/3:00 and 19:00–23:00, due to the low load demand and the absence of PV output, the ESSs are used to discharge the appropriate amount of power to reduce the supply pressure on the main grid and the RoR-SH. From 3:00/4:00 to 18:00, the load demand is higher during this period, the PV output also increases considerably, and the ESS is charged to absorb the excess PV/RoR-SH output to reduce the amount of abandoned light/water. 

In the period of withered water and higher PV generation, as a result of the slight increase in PV output, the reduction output in the RoR-SH, and the reduction in load demand compared to case 1, the ESSs are discharged from 0:00–8:00/9:00 and 18:00/19:00–23:00 and charged from 9:00/10:00–17:00/18:00. 

As shown in Figure 16, in order to increase the capacity of renewable energy consumption and improve the power transfer efficiency, the wide-range TCSC operates in capacitive mode after a certain value of PV generation is reached, prompting the power from the subnetwork side of RoR-SH to be delivered to the side of PV. Additionally, it operates in an inductive model for the rest of the time and facilitates the power flow in the reverse direction.

#### 4.2.3. Superiority Statement

**Figure 17:**
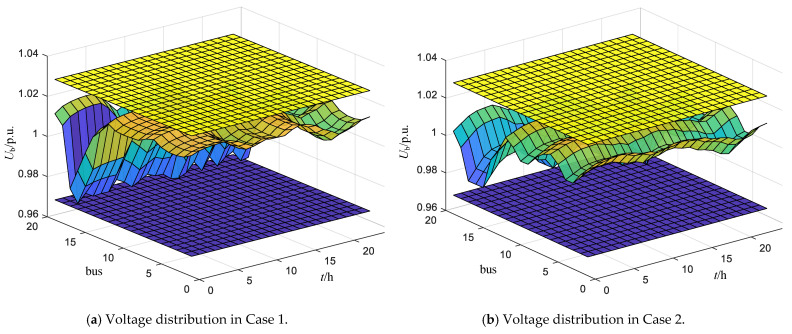
Voltage distribution of all nodes in the proposed ADN.

First, we present the voltage distribution of all nodes in the proposed ADN over a 24-h period for Case 1 and Case 2, as shown in Figure 17. The X-axis represents the ADN node numbers (0–19), the Y-axis represents time (0–24 h), and the Z-axis represents node voltage. The yellow plane above indicates the upper voltage limit (+3%), while the purple plane below represents the lower voltage limit (−3%). The curved surface in the middle shows the voltage distribution at different times for each node. It is evident that all node voltages remain within the voltage constraint range throughout all time periods without exceeding limits. This indicates that the proposed strategy effectively addresses the voltage violation issues in the network of RoR-SH.

**Figure 18:**
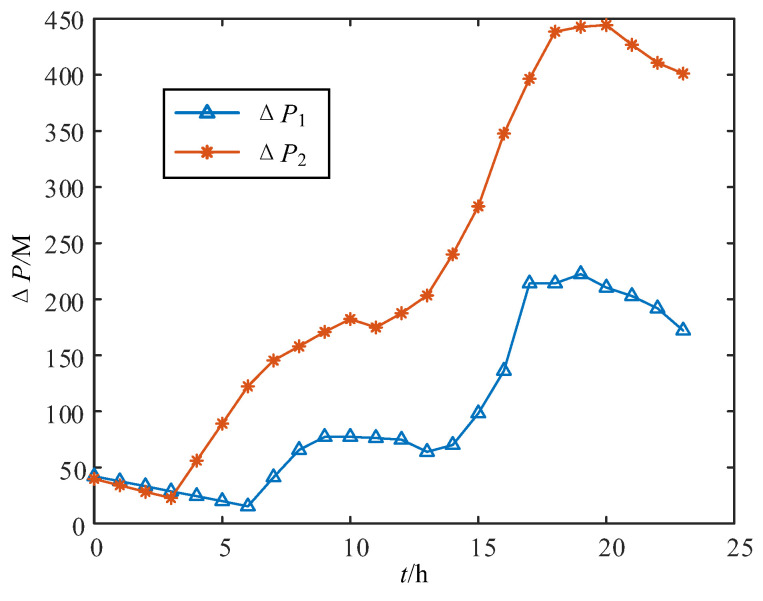
Power difference in Case 1 and Case 2.

Next, calculate the difference in power $\Delta P_{1}$ and $\Delta P_{2}$ at the junctions of the two subnetworks with and without the wide-range TCSC in Case 1 and Case 2, as shown in Figure 18. It can be concluded that the power transmission efficiency of a line equipped with wide-range TCSC is always greater than that of a line without wide-range TCSC. This design offers obvious benefits for ADNs in mountainous areas, where lines are long and the environment is complex.

**Figure 19:**
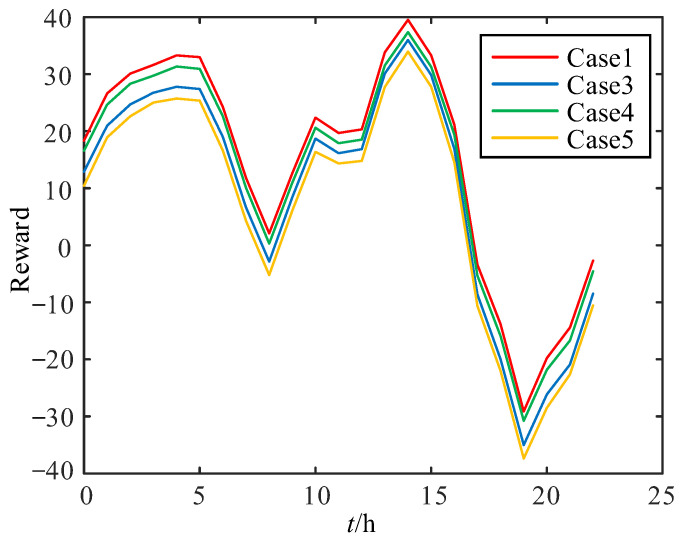
Reward comparison.

In order to verify the advantages of the proposed strategy in renewable energy consumption, reward values of Case 1, Case 3, Case 4, and, Case 5 for each action are compared over 24 h, as shown in Figure 19.

Reward results in Case 3 and Case 4 illustrate that equipping ESS and wide-range TCSC individually also improves the renewable energy consumption capacity of the ADN to a certain extent, in which wide-range TCSC can balance the active power flow among feeders, improve the power delivery efficiency, and reduce line losses, while ESS further improves the load profile to match the renewable energy generation and reduces the amount of light and water rejections. Nevertheless, it can be seen that the best reward value results can only be obtained by equipping both ESS and wide-range TCSC at the same time, indicating that the renewable energy consumption capacity is the strongest in case 1.

## 5. Conclusions

With the wide application of high proportion DG, the development of ADNs in mountainous areas with the characteristic of small-section lines and light loads face great challenges, such as difficulties in optimization operation, light and water abandonment, and voltage violation. To address the above issue, this paper proposes a wide-range TCSC-based ADN in mountainous areas and a corresponding DRL-based optimization operation strategy considering hydropower-photovoltaic-ESS complementarity, where the analysis and the arithmetic examples can lead to the following conclusions.

(1) Theoretical analysis and simulation verification demonstrate that the proposed wide-range TCSC can better achieve the function of power reversal and improve the power transfer efficiency under the steady-state operation environment, which provides the conditions for optimization operation.

Compared with the current research popularity of converter-based soft open point (SOP), which can also achieve this function, the smaller footprint and lower cost of TCSC make it more suitable for the ADN in mountainous areas.

(2) A DRL-based optimization operation strategy is proposed considering hydropower-photovoltaic-ESS complementarity for the proposed ADN. Based on the proposed strategy, digital simulation using the proposed ADN in mountainous areas is conducted. It is verified that the proposed optimal operation strategy, combined with the wide-range TCSC, can effectively enhance the renewable energy consumption capacity and power transfer efficiency of the ADN in mountainous areas while avoiding voltage violation issues. This offers practical value for mountainous distribution networks.

Currently, the equipment in mountainous distribution networks is often quite outdated and lacks the infrastructure to support advanced monitoring and control technologies. These deficiencies need to be addressed. The optimization operation issue of the ADN networks is a comprehensive problem, and more means will be further studied and explored in the future to improve the operation of distribution networks.

## Figures and Tables

**Table 1:** Parameter setting of the prandsed ADN.

Symbol	Value	Symbol	Value
*U*_11_, *U*_21_	220 V	*L*	3.66 × 10^−3^ H
*U*_12_, *U*_22_	110 V	*R*_Load_	3 Ω
*X/R* ratio	7	*α*_L_	132°
*C*_1_	100 × 10^6^ F	$\alpha_{C}$	145.5°
*C*_2_	$501\times 10^{-6}$F	$\alpha_{\mathrm{Lmax}}$	140°
*k*_T_	220/110/10	$\alpha_{\mathrm{Cmin}}$	144°

## Data Availability

Data are unavailable due to privacy.

## Nomenclature

$U_{i}∠\delta_{i}$	Voltage and phase angle of the power supply *i*.
$X_{Gi}$	Internal resistance of the power supply *i*.
$X_{Li}$	Equivalent impedance of the line *i*.
Ba*i*/Bb*i*	Circuit breaker Ba*i*/Bb*i*.
$U_{si}$	Voltage on the low voltage side of the transformer T*i*.
$R_{si}$	Equivalent resistance of the transformer T*i*.
$Z_{Li}$	Line impedance of the line *i* π-equivalent circuit.
$Z_{Ti}$	Winding *i* impedance of the three-winding transformer equivalent circuit.
$Y_{Li}$	Conductance of line i π-equivalent circuit.
$Y_{\mathrm{LT}}$	Conductance of three-winding transformer equivalent circuits.
